# Genotype‐phenotype correlations, dystonia and disease progression in spinocerebellar ataxia type 14

**DOI:** 10.1002/mds.27334

**Published:** 2018-03-30

**Authors:** Viorica Chelban, Sarah Wiethoff, Bjørn K. Fabian‐Jessing, Nourelhoda A. Haridy, Alaa Khan, Stephanie Efthymiou, Esther B. E. Becker, Emer O'Connor, Joshua Hersheson, Katrina Newland, Allan Thomas Hojland, Pernille A. Gregersen, Suzanne G. Lindquist, Michael B. Petersen, Jørgen E. Nielsen, Michael Nielsen, Nicholas W. Wood, Paola Giunti, Henry Houlden

**Affiliations:** ^1^ Department of Molecular Neuroscience University College London, Institute of Neurology London UK; ^2^ National Hospital for Neurology and Neurosurgery London UK; ^3^ Department of Neurology and Neurosurgery Institute of Emergency Medicine Chisinau Republic of Moldova; ^4^ Center for Neurology and Hertie Institute for Clinical Brain Research Eberhard‐Karls‐University Tübingen Germany; ^5^ Department of Clinical Genetics Aalborg University Hospital Aalborg Denmark; ^6^ Department of Neurology and Psychiatry Assiut University Hospital, Faculty of Medicine Assiut Egypt; ^7^ Department of Physiology, Anatomy and Genetics University of Oxford Oxford UK; ^8^ Department of Clinical Genetics Aarhus University Hospital Aarhus Denmark; ^9^ Danish Dementia Research Centre, Neurogenetics Clinic, Department of Neurology, Rigshospitalet University of Copenhagen Copenhagen Denmark; ^10^ Department of Clinical Genetics, Rigshospitalet University of Copenhagen Copenhagen Denmark; ^11^ Department of Neurology Aalborg University Hospital Aalborg Denmark; ^12^ Deparmtent of Molecular Neuroscience Ataxia Centre UCL, Institute of Neurology London UK

**Keywords:** *PRKCG*, SCA14, dystonia, ataxia, genetics

## Abstract

**Background:** Spinocerebellar ataxia type 14 is a rare form of autosomal dominant cerebellar ataxia caused by mutations in protein kinase Cγ gene. Clinically, it presents with a slowly progressive, mainly pure cerebellar ataxia.

**Methods:** Using next generation sequencing, we screened 194 families with autosomal dominant cerebellar ataxia and normal polyglutamine repeats. In‐depth phenotyping was performed using validated clinical rating scales neuroimaging and electrophysiological investigations.

**Results:** We identified 25 individuals from 13 families carrying pathogenic mutations in protein kinase Cγ gene. A total of 10 unique protein kinase Cγ gene mutations have been confirmed of which 5 are novel and 5 were previously described. Our data suggest that the age at onset is highly variable; disease course is slowly progressive and rarely associated with severe disability. However, one third of patients presented with a complex ataxia comprising severe focal and/or task‐induced dystonia, peripheral neuropathy, parkinsonism, myoclonus, and pyramidal syndrome. The most complex phenotype is related to a missense mutation in the catalytic domain in exon 11.

**Conclusion:** We present one of the largest genetically confirmed spinocerebellar ataxia type 14 cohorts contributing novel variants and clinical characterisation. We show that although protein kinase Cγ gene mutations present mainly as slowly progressive pure ataxia, more than a third of cases had a complex phenotype. Overall, our case series extends the phenotype and suggests that protein kinase Cγ gene mutations should be considered in patients with slowly progressive autosomal dominant cerebellar ataxia, particularly when myoclonus, dystonia, or mild cognitive impairment are present in the absence of polyglutamine expansion. © 2018 The Authors. Movement Disorders published by Wiley Periodicals, Inc. on behalf of International Parkinson and Movement Disorder Society.

Autosomal dominant cerebellar ataxias are a heterogeneous group of neurodegenerative disorders. Spinocerebellar ataxia type 14 (SCA14) is a very rare form with an estimated incidence of 1% to 4% of all autosomal dominant cerebellar ataxias.^1^, ^2^ It is caused by mutations in the protein kinase Cγ gene *(PRKCG)*. Missense mutations are responsible for most of SCA14 reported cases but deletions and splicing variants have also been described.^3^, ^4^ The mutations are primarily located in the regulatory domains C1 and C2, with C1 harboring the largest cluster of pathogenic *PRKCG* variants.^5^ *PRKCG* is expressed predominantly in Purkinje cells. Neurodegeneration in SCA14 is thought to result from defective aggregation and disrupted synaptic activity.^6^, ^7^ Clinically, SCA14 presents with slowly progressive, early‐onset, mainly pure cerebellar ataxia.^8^ Some complex ataxic phenotypes have been associated with *PRKCG* mutations, including focal, task‐induced dystonia, myoclonus, and pyramidal syndrome in single cases or small cohorts.^1^, ^9^

We describe the genetic and clinical analysis of one of the largest genetically confirmed SCA14 cohorts contributing novel variants and supporting the phenotypic heterogeneity of *PRKCG* mutations.

## Methods

### Subjects

Proband cases with autosomal dominant, mainly pure, cerebellar ataxia were recruited with informed consents. Most of the families were of European ancestry (n = 194, of which 190 were British, 3 Danish, and 1 Italian). All cases had comprehensive phenotyping performed by neurogenetics specialists. Ataxia was quantified in 17 cases using the validated Scale for Assessment and Rating of Ataxia (SARA).^10^ Pure phenotype was defined as cerebellar syndrome, with brisk reflexes and neurogenic bladder accepted as additional features. Complex phenotype was defined as cases with cerebellar syndrome, plus at least 1 other neurological sign that cannot be explained by associated comorbidities. Disability score was defined as follows: 0 = asymptomatic, 1 = able to walk but difficulty with running, 2 = uses one stick and/or orthosis, 3 = uses 2 sticks/walker, 4 = unable to walk, uses wheelchair.

Results from additional investigations were analyzed where available: neuroimaging with brain magnetic resonance imaging (MRI; n = 12), fluorodeoxyglucose (FDG)‐positron emission tomography (PET) (n = 2), electromyogram or nerve conduction studies (n = 7), and videography. The cases with symptomatic memory/cognitive complaints (n = 5) had formal cognitive assessment performed by qualified neuropsychiatrists.

### Genetic Testing

DNA was extracted and investigated under approval of the joint ethics committee of UCL Institute of Neurology and the National Hospital for Neurology and Neurosurgery, London, UK (UCLH: 04/N034). Families IX and XIII were tested with informed consent as part of a clinical diagnostic evaluation (Centogene AG, Rostock, Germany and Molecular Genetic Laboratory, Department of Clinical Genetics, Rigshospitalet, University of Copenhagen, Copenhagen, Denmark). Apart from 2 families (IX and XIII), the SCA1, 2, 3, 6, 17, Frataxin (*FXN*)‐repeat expansions, Junctophilin 3 (*JPH3*), and common DNA polymerase gamma (*POLG)‐*mutations were excluded in all cases using standard diagnostic techniques. Ataxia‐telangiectasia mutated gene (ATM) kinase activity and levels of radiation‐induced chromosome damage as well as aprataxin and senataxin levels were normal in all complex cases.

In the research approach, a custom‐sequencing panel was designed to amplify the coding exons of *PRKCG* using the Illumina Truseq custom amplicon kit following the manufacturer's protocol. Libraries were prepared according to the standard protocols and then sequenced using an Illumina MiSeq. Bioinformatics analysis included alignment of reads to the hg19 genome build using Novoalign (Novocraft, Selangor, Malaysia) and variant calling using SAMtools (http://samtools.sourceforge.net/). Variant annotation was performed with ANNOVAR,^11^ and coverage metrics were assessed using an in‐house modified Bedtools coverageBed script. After mutation identification, all of the variants reported here were confirmed using Sanger sequencing.

For every identified *PRKCG* mutation, we determined pathogenicity and novelty. Novel and very rare (minor allele frequency < 0.1%), coding/splicing, and heterozygous variants that are predicted to be pathogenic were considered as likely causal variants.^12^, ^13^ For the missense variants, the prediction was based on conservation across different species and assessing novel amino acid changes in known pathogenic positions. Where possible, segregation was confirmed in other family members.

### Statistical Analysis

Two‐tailed *t*‐tests were used to assess the correlation between disease duration and SARA score. *P* ≤ .05 was considered statistically significant.

## Results

### Genetic Analyses

**Figure 1:**
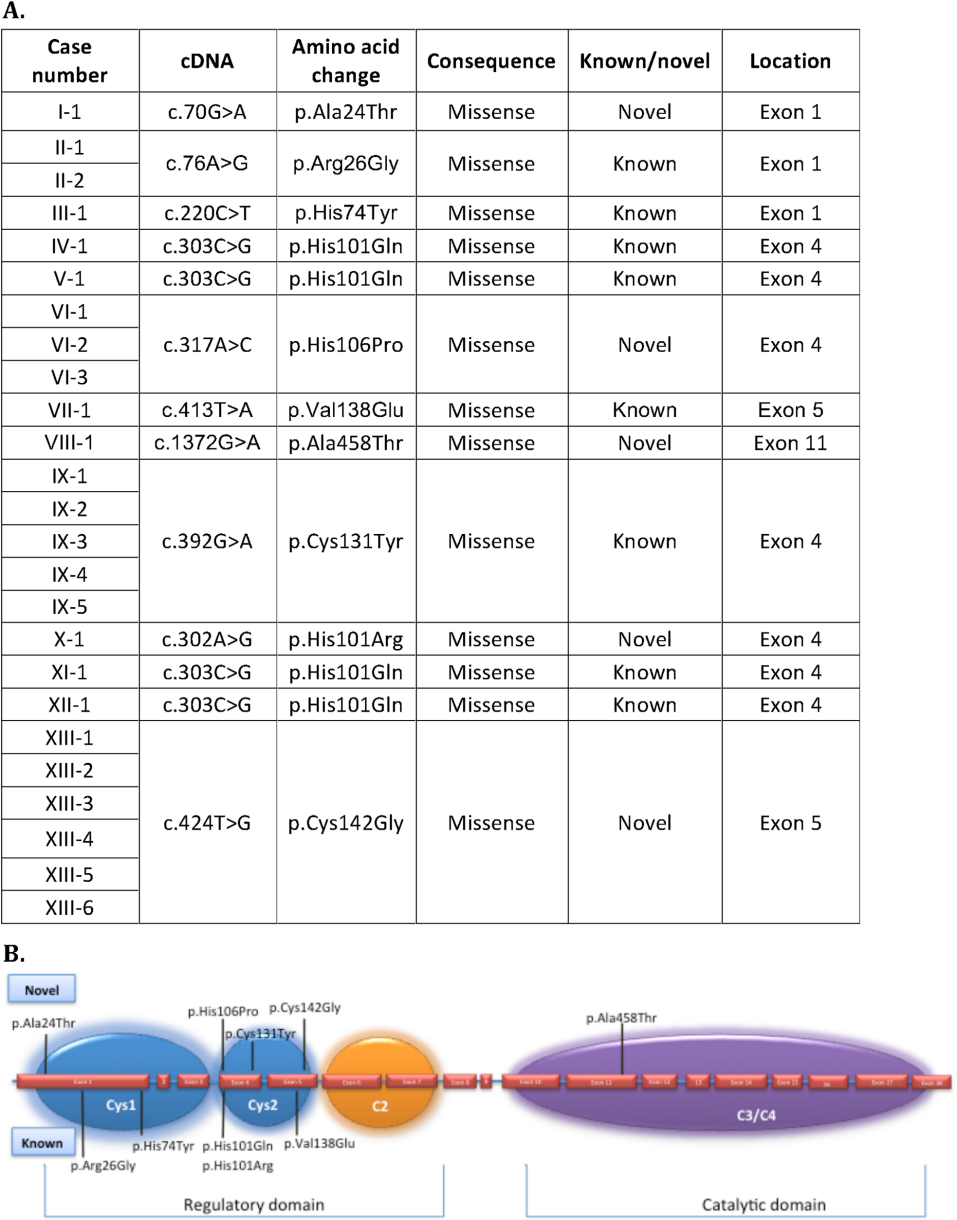
Description of mutations identified in spinocerebellar ataxia type 14 (SCA14) cohort. (**A**) Description of all protein kinase Cγ gene (*PRKCG*) mutations identified in the study (ENST00000263431). (**B**) Schematic representation of the *PRKCG* gene with the mutations identified in our study. The exons are represented approximately to scale. The introns are represented by a blue line between exons. The *PRKCG* protein's regulatory functional domains are indicated: blue = cysteine‐rich region C1 (cys1 and cys2); orange = Ca^2+^ sensitive region (C2); purple = the catalytic domain containing kinase (C3) and substrate recognition (C4) regions. The novel mutations are indicated on the top and the known mutations on the bottom of the figure. [Color figure can be viewed at http://wileyonlinelibrary.com]

From the 194 index cases, a total of 13 pathogenic mutations in *PRKCG* (ENST00000263431) were confirmed. We identified 10 unique mutations, of which 5 were novel and 5 known pathogenic variants (Fig. 1A). A total of 9 mutations were unique to 1 kindred only. The most frequent mutation was the previously reported c.303C>G (p.His101Gln) found in 4 of our families. Three mutations were located in exon 1, 4 mutations in exon 4, and 2 mutations in exon 5 as part of the regulatory domains C1a and C1b. One mutation was located in the catalytic domain in exon 11 of *PRKCG* (Fig. 1B). All variants were missense mutations.

**Figure 2:**
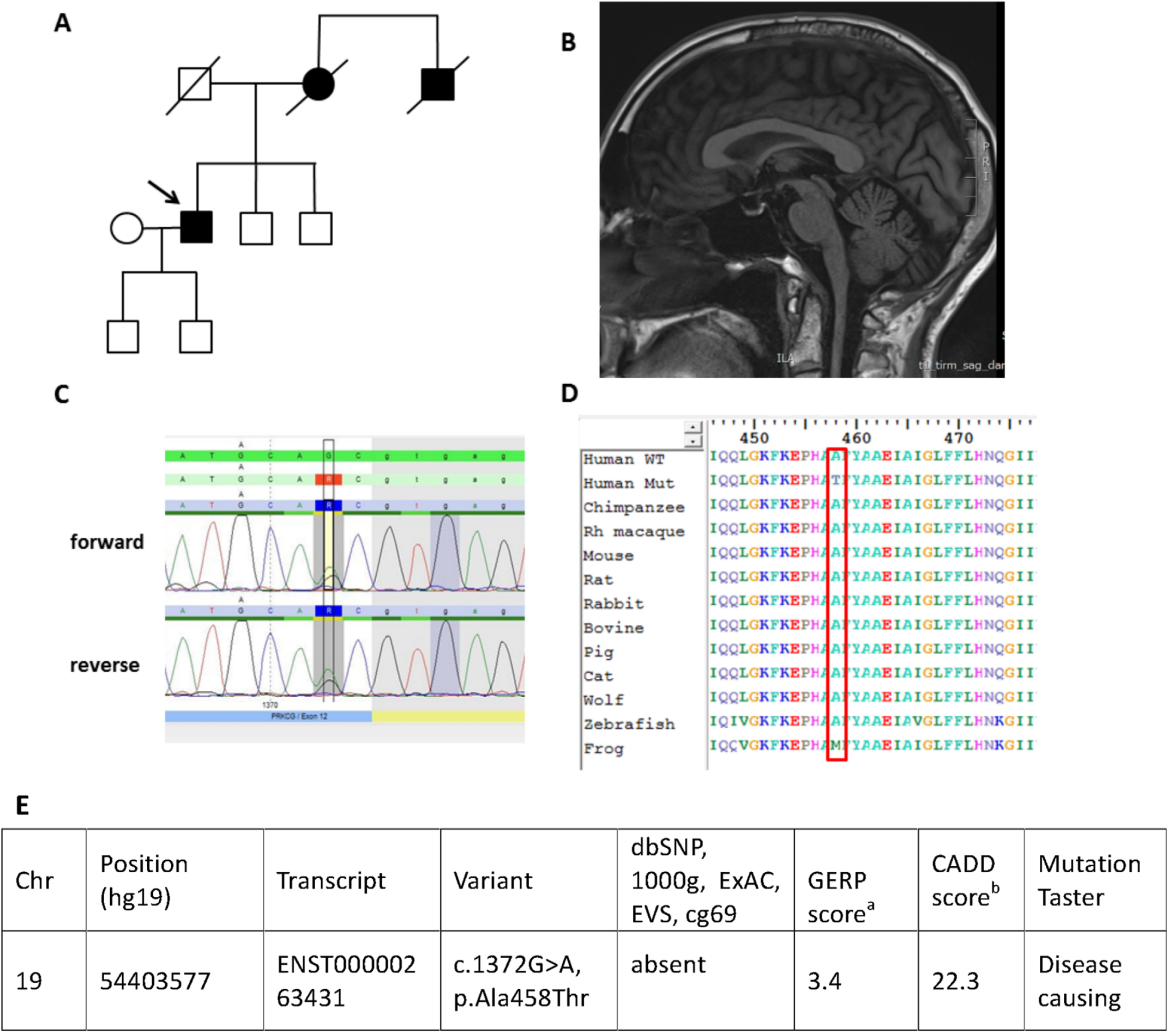
Genetic and clinical details of case VIII‐1 presenting with an unusual phenotype of severe dystonia and peripheral neuropathy. **(A**) Family tree. (**B**) Sagittal MRI of patient at age 65 showing mild cerebellar atrophy and thin cervical cord. (**C**) Sanger sequencing result of the novel mutation c.1372G>A. (**D**) Conservation through species of the novel mutation c.1372G>A. (**E**) Frequency and in silico predictions of the heterozygous, novel missense protein kinase Cγ gene (*PRKCG*) mutation identified. ^a^GERP, positive conservation scores represent a substitution deficit and indicate that a site may be under evolutionary constraint. Negative scores indicate that a site is probably evolving neutrally. Positive scores scale with the level of constraint, such that the greater the score, the greater the level of evolutionary constraint inferred to be acting on that site. ^b^CADD, Combined Annotation Dependent Depletion is a tool for scoring the deleteriousness of single nucleotide variants as well as insertion/deletions variants in the human genome across a wide range of functional categories, effect sizes, and genetic architectures. GERP, Genomic Evolutionary Rate Profiling; D, deleterious/damaging/disease‐causing. [Color figure can be viewed at http://wileyonlinelibrary.com]

Case VIII‐1 had a novel heterozygous mutation c.1372G>A (p.Ala458Thr) confirmed by Sanger sequencing (Fig. 2A‐C). This mutation is located at a highly conserved amino acid position (Fig. 2D). In silico tools predict disease‐causing effects (Mutation Taster; http://www.mutationtaster.org/) and a highly likely protein function interference (Align‐GVD, http://agvgd.iarc.fr/) with a Combined Annotation Dependent Depletion (http://cadd.gs.washington.edu/home) score of 22.3 and a Genomic Evolutionary Rate Profiling score of 3.4 (Fig. 2E). No other family members were available for further testing.

Family XIII is a large Danish family with 6 affected individuals. As part of a clinical diagnostic evaluation, a novel heterozygous mutation in exon 5, the c.424T>G (p.Cys142Gly) was identified. The mutation fully segregated with the disease in the family. This mutation is absent from all publicly available databases (1000 Genomes Project, http://www.1000genomes.org 14; National Heart, Lung, and Blood Institute "Grand Opportunity" (NHLBI GO) Exome Sequencing, http://evs.gs.washington.edu 15; and Exome Aggregation Consortium, http://exac.broadinstitute.org/ 16) and is predicted to affect the Cys1 regulatory domain.

Case I‐1 has a novel heterozygous change c.70A>G (p.Ala24Thr) that segregated with disease in the family. This change is located in a highly conserved amino acid position in the regulatory C1a domain. Family VI and family X presented novel amino acid changes in known pathogenic positions c.317A>C (p.His106Pro) and c.302A>G (p.His101Arg), respectively.

The other cases had previously described pathogenic mutations in *PRKCG* that segregated with the disease.

### Clinical Summary

**Figure 3:**
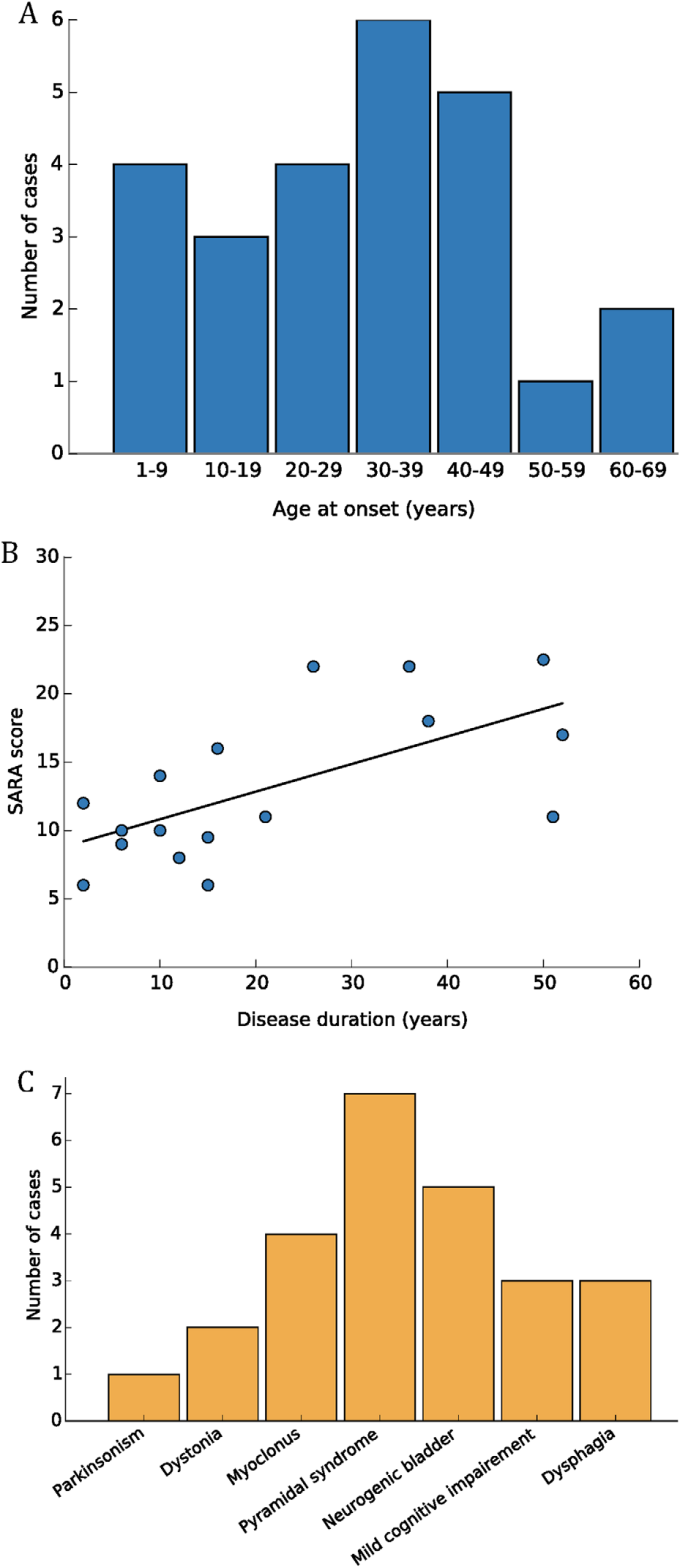
Genotype‐phenotype correlations in spinocerebellar ataxia type 14 (SCA14). **(A**) Age of onset in an SCA14 cohort. (**B**) Correlation between Scale for Assessment and Rating of Ataxia (SARA) score and disease duration. (**C**) Complex phenotypes in SCA14. [Color figure can be viewed at http://wileyonlinelibrary.com]

SCA14 individuals included in our cohort presented with variable disease onset and phenotype. The mean age of onset was 30.6 years with a range from 3 to 66 years, revealing a high variability between and within the families. Within families, an earlier onset was observed in the offspring of affected parents; however, we attribute this to recall and ascertain bias. An early age of onset (defined as onset before 20 years of age) was identified in 40% of cases. However, a late onset (after the age of 50) was reported in 12% of cases (3/25; Fig. 3A). Mean disease duration was 18 years (range 1‐52 years). The mean SARA score at the last examination (for 17 cases with a SARA score) was 13.1, consistent with moderate severity. SARA scores correlated positively with disease duration (*P* = .02; Fig. 3B), but not with age of onset. Disease onset was related to gait or limb ataxia. Table 1 presents the results of detailed clinical examination. Screening for atypical features revealed a complex ataxia phenotype in 36% (9/25) of cases presenting with ataxia and 1 additional neurological sign or combination of signs, including severe focal dystonia, task‐induced dystonia, severe peripheral neuropathy, parkinsonism, myoclonus, tremor, and pyramidal syndrome. Importantly, cases VIII‐1 and II‐2 presented with the most complex phenotypes (Supporting Information Videos 1 and 2).

**Table 1:** Description of all genetically diagnosed spinocerebellar ataxia type 14 phenotypes

Case number/phenotype	I‐1	II‐1	II‐2	III‐1	IV‐1	V‐1	VI‐1	VI‐2	VI‐3	VII‐1	VIII‐1	IX‐1	IX‐2	IX‐3	IX‐4	IX‐5
Age at onset (years)	40	7	20	20	64	18	12	20	34	18	35	66	46	56	42	33
Symptom at onset	Gait ataxia	Gait ataxia	UL and LL ataxia	Gait ataxia	Gait ataxia	Gait ataxia	UL and LL ataxia	Gait ataxia	Gait ataxia	Gait ataxia	Gait ataxia	Gait/balance problems/ dysartria/limb ataxia	UL and LL ataxia	Gait problems/limb ataxia	Balance impairment	Gait ataxia
Disease duration	10	36	38	15	16	51	50	12	10	52	26	2	23	2	21	2
SARA score	10	22	18	6	16	11	22.5	8	14	17	22	NA	NA	NA	11	6
Disability	1	3	2	0	2	2	3	2	2	1	2	1	3	1	1	1
Phenotype	Pure	Pure	Complex	Pure	Pure	Pure	Pure	Pure	Pure	Complex	Complex	Pure	Pure	Pure	Pure	Complex
Limb ataxia	+	+	+	+	+	+	+	+	+	+	+	+	+	+	+	+
Eye signs	Nystagmus	Nystagmus	Nystagmus	Nystagmus	Nystagmus	−	Nystagmus	Nystagmus	Nystagmus	Nystagmus	Nystagmus	Saccadic pursuit	Nystagmus	Saccadic pursuit	Broken‐up pursuit	Broken‐up pursuit
Dysarthria	+	+	+	+	+	+	+	+	+	+	+	+	+	−	+	+
Pyramidal syndrome	−	−	+	−	−	−	+	+	+	−	+	−	−	−	−	−
Parkinsonism	−	−	Gait freezing, bradykinesia	−	−	−	−	−	−	−	−	−	−	−	−	−
Dystonia	−	−	−	−	−	−	−	−	−	Writer's cramp	Dystonia, severe, laterocolis and limb dystonia	−	−	−	−	−
Myoclonus	−	−	Mild, peri‐oral facial myoclonus	−	−	−	−	−	−	−	−	−	−	−	−	Facial minimyoclonus and slight finger/arm myoclonus
Peripheral neuropathy	−	−	−	−	−	−	−	−	−	−	Severe neuropathy in the LL	−	−	−	−	−
Other	Mild dysphagia	−	Neurogenic bladder	−	−	−	Neurogenic bladder	Mild dysphagia	Mild dysphagia	−	Neurogenic bladder	Hearing impairment, Increased reflexes, clonus	Decreased reflexes UL, Increased reflexes in LL	Increased reflexes LL	Brain PET FDG: reduced metabolic activity cerebellum	Brain PET FDG: reduced metabolic activity cerebellum
Cerebellar atrophy on MRI	Mild	Moderate	Moderate	Mild	NA	Mild	Moderate	NA	NA	Mild	Moderate	NA	NA	Moderate	Mild	Mild
NCS/EMG	NA	Normal	Normal	Normal	NA	Normal	Normal	NA	NA	Normal	Severe axonal length dependent neuropathy	NA	NA	NA	NA	NA
Neuropsychology	NA	NA	Mild cognitive dysfunction affecting anterior and subcortical functions	NA	NA	NA	NA	NA	NA	NA	NA	NA	NA	NA	NA	Normal

Case number/ Phenotype	X	XI	XII	XIII‐1	XIII‐2	XIII‐3	XIII‐4	XIII‐5	XIII‐6
Age at onset (years)	31	43	28	39	48	6	6	30	3
Symptom at onset	Gait ataxia	Gait ataxia	Gait ataxia	Gait and balance impairment	Gait impairment	Upper limbs postural tremor followed by gait and balance impairment	Limb ataxia	Balance impairment/tremor/limb ataxia	Limb ataxia
Disease duration	12	8	12	15	6	51	23	6	1
SARA score	NA	NA	NA	9.5	10	12	NA	9	NA
Disability	2	2	2	1	1	1	1	1	1
Phenotype	Pure	Pure	Pure	Complex	Complex	Complex	Pure	Complex	Complex
Limb ataxia	+	+	+	+	+	+	+	+	+
Eye signs	Nystagmus	Nystagmus	Nystagmus	Broken‐up pursuit	Unstable pursuit	Broken‐up pursuit	Slow pursuit	Broken‐up pursuit	−
Dysarthria	+	+	+	+	+	+	−	+	+
Pyramidal syndrome	−	−	−	+	+	−	−	−	−
Parkinsonism	−	−	−	−	−	−	−	−	−
Dystonia	−	−	−	−	−	−	−	−	−
Myoclonus	−	−	−	−	−	−	−	−	Mild, occasional myoclonic truncal jerks
Peripheral neuropathy	−	−	−	−	−	−	−	−	−
Other	−	−	−	−	Urge incontinence, mild impairment of vibration sense	Urge incontinence. Decreased reflexes UL and LL, postural hand and head tremor	Increased patellar reflexes	Increased reflexes UL, postural hand tremor	−
Cerebellar atrophy on MRI	NA	NA	NA	NA	NA	NA	Mild	NA	NA
NCS/EMG	NA	NA	NA	NA	NA	NA	NA	NA	NA
Neuropsychology	NA	NA	NA	NA	NA	Cognitive dysfunction affecting concentration and memory	Decreased memory and concentration	Normal	NA

EMG, electromyogram; NA, not available; + , present; ‐, absent; SARA score, Scale for Assessment and Rating of Ataxia score; LL, lower limb; UL, upper limb; NCS, nerve conduction studies.

#### Complex Phenotypes in SCA14

Case VIII‐1 developed a slowly progressive ataxia with onset in the mid‐30s initially affecting dexterity and gait. Cerebellar ataxia was associated with dysarthria, mild neurogenic bladder, slight forgetfulness, and clumsiness of hands. At the age of 55 years, he noted new intermittent shaking of his head. This dystonic head tremor rapidly progressed toward severe, constant dystonic posturing of the head and right hand. Currently, his clinical findings consist of dystonic head tremor with constant titubation of head at rest, laterocollis to the left, and subsequent hypertrophy of the right sternocleidomastoid associated with the underlying progressive cerebellar syndrome. A bilateral mild wasting of the small hand muscles is noted together with absent reflexes and severe peripheral neuropathy in the lower limbs (Supporting Information Video 1). Nerve conduction studies showed a severe length‐dependent axonal sensory motor neuropathy and a muscle biopsy was suggestive of neurogenic changes only. The causes of acquired peripheral neuropathy had been excluded. The brain MRI showed cerebellar hemispheric and vermis atrophy (Fig. 2B). Neuropsychometry testing revealed mild cognitive dysfunction affecting the anterior and subcortical functions. Formal neuropsychometry testing was also performed in 3 other individuals from a large SCA14 family (XIII‐3, XIII‐4, and XIII‐5) as well as case IX‐5. The 2 affected individuals (XIII‐3 and XIII‐4) also reported cognitive dysfunction with difficulties in concentration and memory. Case XIII‐3 is 57 years old and case XIII‐4 is 29 years old. No other cases had cognitive problems identified or reported during clinical examination and follow‐up.

The second case presenting with focal dystonia (case VII‐1) had a slowly progressive ataxic syndrome associated with “writer's cramp,” currently treated with regular Botox injections.

Case II‐2 is a member of a large family that we have described previously.^17^ He presented with gait and limb ataxia when aged in his 20s. Around the same time, he noticed problems initiating movements, such as walking over a doorstep. The disease slowly progressed and 38 years later he presented with moderate ataxia (SARA score 18) associated with pyramidal syndrome, parkinsonism (Supporting Information Video 2), mild rest, spontaneous, peri‐oral myoclonus, and neurogenic bladder. Interestingly, his family member (II‐1) presents with pure cerebellar ataxia demonstrating evident intrafamilial heterogeneity.

One frequently associated sign in the complex phenotypes was myoclonus. Myoclonus was present in 12% of cases (3/25). This was clinically characterized as occasional and mild, spontaneous myoclonus, facial peri‐oral in the case II‐2; as facial mini‐myoclonus with slight finger/arm myoclonus in case X‐5; and as occasional truncal jerks in case XIII‐6. The myoclonus was identified within the first 2 years from disease onset (cases X‐5 and XIII‐6) and remained mild in the case with almost 40 years of disease duration (case II‐2). In all cases, the mild severity of myoclonus did not mandate any treatment, and no further electrophysiological investigations were performed.

A quarter of our cases had an associated pyramidal syndrome in the lower limbs. Neurogenic bladder was also present in these patients. In addition, 3 patients presented with mild dysphagia. The other two thirds of cases presented with pure cerebellar ataxia of variable severity (Table 1).

MRI of the brain was available in 14 cases and showed only mild to moderate cerebellar atrophy in all cases. Two individuals had brain positron emission tomography with 2‐deoxy‐2‐[fluorine‐18]fluoro‐D‐glucose integrated with computed tomography that showed reduced FDG metabolic activity in the cerebellum.

## Discussion

We studied a large cohort (n = 194) of mainly pure, autosomal dominant cerebellar ataxia cohort negative for trinucleotide repeats and identified 13 families with disease‐causing mutations in *PRKCG* with a variable spectrum of disease phenotype and severity. Our data suggests that *PRKCG* mutations have a frequency of 6.7% (13/194). Previous studies showed SCA14 frequencies varying 1.5% to 7.5% in different ethnic groups.^1^, ^2^, ^3^ Also, early studies screened only parts of the gene, therefore some mutations may have been missed.^2^

In the 13 families with *PRKCG* mutations, we identified 10 unique pathogenic variants suggesting that recurrent *PRKCG* mutations are infrequent. All mutations are missense variants and are located within a functional domain. The majority of mutations are located in the regulatory C1 domain, consistent with previous reported data. The exact mechanism leading to disease in SCA14 is still unknown, and the simplistic loss‐of‐function hypothesis would not fully explain the autosomal dominant inheritance in the context of the Protein kinase C gama (PKCγ) knockout mouse that had only a mild ataxia and did not show any morphological changes in the cerebellum.^18^, ^19^ In vivo experiments show that mutations in the C1 domain decrease membrane binding, membrane retention time, and substrate phosphorylation, resulting in sustained high levels of intracellular Ca^2+^ upon cell stimulation and aberrant intracellular signalling.^20^ However, mutations in the catalytic domain showed an exaggerated inhibitory effect of Ca^2+^ entry in mutant cells when compared with wild type and Ca^2+^ influx was completely blocked.^20^ Therefore, it is becoming more clear that both sustained high Ca^2+^ influx as well as inhibition of Ca^2+^ influx is involved in SCA14 pathology depending on the functional domain that is affected. Interestingly, it was shown recently that mutations in the catalytic but not in the regulatory domain also show inhibition of the dendritic development in addition to a different pattern of the enzymatic activity.^21^ These findings together with the difference in PKCγ biological activity both in vitro and in vivo cell assays show that mutations located in different functional domains lead to disease through different mechanisms with a mutation‐specific SCA14‐resulting phenotype.^20^, ^22^, ^23^ Although less frequent, mutations in the catalytic domain have been described to cause a more complicated SCA14.^2^, ^20^, ^24^ Clinically, these mutations have been associated with a wider age of onset (childhood until late 60s) and a more complex phenotype including higher rate of cognitive dysfunction, myoclonus, dystonia, and reduced vibration sense (Table 2). In our study, the genotype‐phenotype correlation confirms that the novel missense variant c.1372G>A (p.Ala458Thr) located in the catalytic domain produced the most complex and severe phenotype. We would like to highlight the unusual presentation of ataxia associated with a severe, time‐deferred dystonic component and a severe peripheral neuropathy in the context of the missense p.Ala458Thr. Dystonia has been previously reported in SCA14 mainly described as focal, task‐induced dystonia.^9^ Our case series extends the phenotype and supports the association of ataxia and dystonia in SCA14. To our knowledge, severe peripheral neuropathy has not been previously formally associated with SCA14; however, several cases of reduced vibration loss were reported in mutations affecting the regulatory domain (Table 2). The association with severe peripheral neuropathy needs to be confirmed in larger cohorts.

**Table 2:** Description of all reported mutations affecting the catalytic domain of protein kinase Cγ gene (*PRKCG*) with the genotype‐phenotype correlation

*PRKCG* mutations	p.Gly360Ser^2^	p.Ser361Gly^3^	p.Ala458Thr (this study)	p.Asp480Tyr^31^	p.Phe643Leu^2^, ^24^	p.Arg659Ser^32^	p.Val692Gly^2^	p.Met697Ile^33^	p.Val177fs^31^
Type of mutation	Missense	Missense	Missense	Missense	Missense	Missense	Missense	102 base pair deletion	1717 base pair deletion
Conservation	Highly conserved	Highly conserved	Highly conserved	Highly conserved	Highly conserved	Highly conserved	Highly conserved	Highly conserved	Highly conserved
Zygosity	Heterozygous	Heterozygous	Heterozygous	Homozygous	Heterozygous	Heterozygous	Heterozygous	Heterozygous/Homozygous	Heterozygous
Penetrance	100%	100%	100%	100%	100%	100%	100%	100%	100%
Age of onset (years)	53	5‐60	35	NA	Childhood to 60	Not available	20	7‐60	Not available
Symptom at onset	Ataxia	Ataxia	Ataxia	Ataxia	Ataxia	Retinitis pigmentosa	Ataxia	Ataxia	Ataxia
Clinical syndrome	Complex, slowly progressive cerebellar ataxia	Slowly progressive cerebellar ataxia	Complex, slowly progressive cerebellar ataxia	Complex, slowly progressive cerebellar ataxia	Complex, slowly progressive cerebellar ataxia	Not available	Slowly progressive cerebellar ataxia	Complex, slowly progressive cerebellar ataxia	Complex, slowly progressive cerebellar ataxia
Associated features	Rippling in small hand muscles, swallowing difficulties	Depression	Dystonic head tremor with constant titubation of head at rest, laterocollis, severe peripheral neuropathy	Intellectual disability	Cognitive decline (8 cases), myokimia (4 cases), diffuse myoclonus in the limbs (1 case), reduced vibration sense in lower limbs (4 cases), chorea in the hands and head tremor (2 cases)	Not available	Decreased vibration sense	Generalised truncal and limb myoclonus	Intellectual disability
Functional analysis and consequence of mutation	Increased kinase activity, even in the absence of activators.^20^ Normal dendritic development^21^	Increased kinase activity within Purkinje cells. Inhibition of dendritic development^20^, ^34^	Not available	Not available	Increased kinase activity within Purkinje cells. Inhibition of dendritic development^20^, ^21^	Not available	Patterns of kinase activity similar to those of the wild‐type enzyme^20^	Increased kinase activity compared to wild‐type^33^	Not available

Our data suggest that in the majority of cases, SCA14 has an onset from childhood to early adulthood, is slowly progressive, and is rarely associated with severe disability, consistent with previous reports.^25^ However, about 12% of cases presented with a late‐onset cerebellar ataxia (>50 years old). The most common genetic conditions with similar presentation in this age group are usually caused by CAG repeat expansions SCAs (SCA 1, 2, 3, and 6) and Friedreich's ataxia that require different approaches for molecular diagnosis. Importantly, although our cohort had a long mean disease duration (18 years), the mean SARA score was consistent with moderate severity, supporting a slow disease progression course. The most severe SARA score was 22.5 in a patient who had the disease for more than 50 years. Therefore, an autosomal dominant cerebellar ataxia with normal SCA repeats, especially when slow progression and predominantly pure phenotype is predominant, should alert the physician to test for *PRKCG* mutations. We show that next generation sequencing can further contribute to diagnosis in cases of late‐onset cerebellar ataxia where the repeats are within normal ranges.

More than a third of the patients in our study presented with a complex ataxia phenotype composing of severe focal and/or task‐induced dystonia, severe peripheral neuropathy, parkinsonism, myoclonus, tremor, and/or pyramidal syndrome. This is consistent with previous reports of complex cases of ataxia associated with dystonia, myoclonus, tremor, and pyramidal syndrome secondary to *PRKCG* mutations.^3^, ^9^, ^26^ In our cohort, myoclonus was identified in 3 cases. This was clinically characterized as occasional and mild, not requiring any treatment. Myoclonus was identified early in the disease and remained mild in the advanced cases. Unfortunately, none of the patients reported having undergone electrophysiological recording, and we are unable to comment further on this aspect; therefore, further studies need to assess myoclonus as it may be more frequent than previously reported.

We report 1 case with a combination of mild parkinsonism (presenting a stooped posture, gait freeing, rigidity, and bradykinesia) in addition to cerebellar ataxia. Interestingly, the rat animal model of *PRKCG* also exhibits a parkinsonian phenotype.^27^ Parkinsonism is rare and mild in SCA14.^9^, ^24^ Our case as well as the Dutch^9^ and French families^24^ presented with subtle parkinsonian syndrome in their early 20s that has not progressed with the disease and did not require Levodopa treatment. The combination of ataxia and parkinsonism is frequent in neurodegenerative ataxias^28^; however, the combination of autosomal dominant SCA and parkinsonism is mainly observed in a parkinsonian form of SCA3 and sometimes in SCA2,^28^, ^29^ where the parkinsonism is present early in the disease. In such cases, clinical differential diagnosis is challenging and only genetic investigation can provide a definite diagnosis. The sporadic neurodegenerative cerebellar ataxias with parkinsonism, such as multiple system atrophy, present usually with a late‐onset and a faster disease progression and are associated with autonomic dysfunction.

In addition, cognitive impairment is only rarely reported in SCA14, and if cognition is affected, only minimal severity is reported,^30^ which was mirrored in our series. However, a formal neuropsychology assessment was conducted in symptomatic cases only limiting our ability to characterize this further. We would like to stress that all SCA14 cases that reported cognitive impairment were before the age of 50 years, and this complaint is not attributed to age‐related associated disorders at present. Cognitive impairment has been reported in other genetic forms of SCA and is not only restricted to SCA14^21^; our study and previous reports warrant a further uniformed and systematic evaluation to validate these results.

Therefore, our data suggests that SCA14 diagnosis in complex phenotypes should be considered in patients with slowly progressive autosomal dominant cerebellar ataxias, particularly when myoclonus, dystonia, or cognitive impairment is present in the absence of polyglutamine expansions. Furthermore, a very mild severity and a slow progression of the additional features such as parkinsonism and myoclonus in SCA14 can help differentiate them from other forms of sporadic neurodegenerative disorders, such as multiple system atrophy (MSA).

## Conclusion

We present one of the largest SCA14 cohorts of patients reported contributing with novel variants and supporting the distinct phenotype spectrum with specific cellular defects resulting from different types of *PRKCG* mutations. Our cases extend the phenotype and support the association of ataxia and severe dystonia in SCA14. The association with severe peripheral neuropathy needs confirmation in larger cohorts.

## Financial disclosures of all authors (for the preceding 12 months)

V.C. is supported by the Association of British Neurologists/MSA Trust Clinical Research Training fellowship (F84 ABN 540868). S.W. is supported by a Brain Research Trust (BRT)‐studentship and the Ministry of Science, Research and the Arts of Baden‐Württemberg and the European Social Fund of Baden‐Württemberg (31‐7635 41/67/1). B.K.F.‐J. was supported by a research grant from Aalborg University Hospital. P.G. works at University College London Hospitals/University College London, which receives a proportion of funding from the Department of Health's National Institute for Health Research Biomedical Research Center's funding scheme, and receives support from the National Institute for Health Research Clinical Research Network.

### Author Roles

(1) Research Project: A. Conception, B. Organization, C. Execution; (2) Statistical Analysis: A. Design, B. Execution, C. Review and Critique; (3) Manuscript Preparation: A. Writing of the First Draft, B. Review and Critique.

V.C.: 1A, 1B, 1C, 2A, 2B, 3A

S.W.: 3A, 3B, 1A, 1C

B.K.F.: 1A, 1B, 1C, 3B

N.H.: 1C, 3B

A.K.: 1C, 3B

S.E. : 1C, 3B

E.B.: 1C, 3B

E.O.: 1C, 3B

J.H.: 1C, 3B

K.N.: 1C, 3B

A.T.H.: 1C, 3B

P.A.G.: 1B, 1C, 3B

S.G.L.: 1C, 3B

M.B.P.: 1B, 1C, 3B

J.E.N.: 1A, 1B, 1C, 3B

M.N.: 1B, 1C, 3B

N.W.: 1A, 1B, 1C, 3B

P.G.: 1A, 1B, 3B

H.H.: 1A, 1B, 3B

## Supporting information

Additional Supporting Information may be found in the online version of this article at the publisher's website.

Supplementary Information 1

Supplementary Information 2
